# The Role of Anterior Cervical Discectomy and Fusion on Relieving Axial Neck Pain in Patients With Single-Level Disease: A Systematic Review and Meta-Analysis

**DOI:** 10.1177/2192568219837923

**Published:** 2019-03-25

**Authors:** Colby Oitment, Tracy Watson, Victor Lam, Mohammed Aref, Alex Koziarz, Edward Kachur, Jetan H. Badhiwala, Saleh A. Almenawer, Aleksa Cenic

**Affiliations:** 1 McMaster University, Hamilton, Ontario, Canada; 2 University of Western Ontario, London, Ontario, Canada; 3 University of Toronto, Toronto, Ontario, Canada

**Keywords:** ACDF, anterior cervical decompression and fusion, axial neck pain, Visual Analogue Scale, Neck Disability Index

## Abstract

**Study Design:**

Systematic review and meta-analysis.

**Objectives:**

This study aims to evaluate the effects of anterior cervical decompression and fusion (ACDF) on axial neck pain in adult patients receiving surgery for myelopathy, radiculopathy, or a combination of both.

**Methods:**

Two independent reviewers completed a librarian-assisted search of 4 databases. Visual Analogue Scale (VAS) and Neck Disability Index (NDI) scores were extracted preoperatively and at 3, 6, 12, 24, 36, 48, and 48+ months postoperatively for ACDF groups and pooled using a random-effects model.

**Results:**

Of 17 850 eligible studies, 37 were included for analysis, totaling 2138 patients analyzed with VAS and 2477 with NDI score. Individual VAS mean differences were reduced at 6 weeks (−2.5 [95% confidence interval (CI): −3.5 to −1.6]), 3 months (−2.9 [−3.7 to −2.2]), 6 months (−3.2 [−3.9 to −2.6]), 12 months (−3.7 [−4.3 to −3.1]), 24 months (−4.0 [−4.4 to −3.5]), 48 months (−4.6 [−5.5 to −3.8]), and >48 months (−4.7 [−5.8 to −3.6]) follow-up (*P* < .0001 for all endpoints). Individual NDI mean differences were reduced at 6 weeks (−26.7 [−30.9 to −22.6]), 3 months (−29.8 [−32.7 to −26.8]), 6 months (−31.2 [−35.5 to −26.8)], 12 months (−29.3 [−33.2 to −25.4]), 24 months (−28.9 [−32.6 to −25.2]), 48 months (−33.1 [−37.4 to −28.7]), and >48 months (−37.6 [−45.9 to −29.3]) follow-up (*P* < .0001 for all endpoints).

**Conclusions:**

ACDF is associated with a significant reduction in axial neck pain compared with preoperative values in patients being treated specifically for myelopathy or radiculopathy. This influences the preoperative discussions surgeons may have with patients regarding their expectations for surgery. The effects seen are stable over time and represent a clinically significant reduction in axial neck pain.

## Introduction

Degenerative cervical disc disease may result in disc herniations, which compress nerve roots causing radiculopathy or compress the spinal cord causing myelopathy.^1^ Patients with degenerative cervical disc disease classically have a significant degree of axial neck pain.^1^ The source of axial neck pain, whether it be discogenic, osseous, muscular, or alignment related, is generally difficult to elucidate on history and physical examination. For this reason, surgeons rely on neurological signs and symptoms to guide treatment.

Anterior cervical discectomy and fusion (ACDF) is a procedure that may be performed for patients with degenerative spondylitic myelopathy or radiculopathy. While the specific indications for surgery vary depending on patient symptoms and anatomic considerations such as previous surgery or deformity, there are well-recognized guidelines for treatment.^1^ Myelopathy guidelines generally recommend decompression for moderate or severe patient symptoms, and the literature suggests surgical decompression for radiculopathy when it is unresponsive to a minimum of 6 weeks of conservative therapy or symptoms are progressive.^1^ Accurate patient understanding of the expected surgical outcomes may improve the perceived success of the surgery and reduce medicolegal litigation.^12^ This involves accurately addressing both patient and surgeon expectations of the procedure preoperatively. While myelopathy may improve with surgery, the results are variable so the general goals of the procedure are to halt progression.^15,16^ Radiculopathy, however, tends to improve more reliably so patients anticipate reliable reductions in arm pain.^2,3^

While axial neck pain symptoms are particularly troubling to many patients, the effects of ACDF on axial neck pain are more poorly understood. Whereas several studies report a reduction in axial neck pain following ACDF, there have been no meta-analyses to date examining the effects of ACDF on cervical spondylitic myelopathy and/or radiculopathy. Accordingly, preoperative discussions may lack clarity regarding the outcomes of surgery with regard to the prognosis of axial neck pain. Therefore, the purpose of this meta-analysis is to examine all randomized controlled trials and prospective cohort studies that recorded preoperative and postoperative axial neck pain scores in order to examine the effect of single-level ACDF on axial neck pain in patients with myelopathy and/or radiculopathy.

## Methods

### Eligibility Criteria

This systematic review included adult patients receiving ACDF. The inclusion criteria for this review were the following: (1) published online and in the English language; (2) randomized controlled trial or cohort study; (3) included preoperative and postoperative data on subjective midline axial neck pain scores (either Visual Analog Scale [VAS] or Neck Disability Index [NDI] scores); (4) single-level pathology. All studies involving corpectomy or multilevel anterior decompressions, as well as posterior decompressive procedures, were excluded. In multiple cases there were studies published with what, in the authors’ best judgement, appeared to be the same ACDF data as a previously published control arm for disc arthroplasty versus ACDF trials. In these cases, the most recent data was utilized and the earlier studies by these authors were not included.

### Search Strategy

Two independent authors searched MEDLINE, EMBASE, PubMed, and Cochrane databases with the guidance of a professional librarian using the following terms: “anterior cervical decompression and fusion” OR “ACDF,” OR “cervical decompression” AND “axial neck pain” OR “midline neck pain” to broaden the number of studies retrieved. Initial search results were vetted for duplicates and a title screen was performed for relevance. Reference lists of eligible studies were screened for additional studies meeting eligibility criteria. Discrepancy was resolved with discussion and consultation with the senior supervising author.

### Risk of Bias

The risk of bias of included studies was assessed by using the Methodological Index for Non-Randomized Studies (MINORS) scale. The MINORS scale scores vary from 0 to 24 for risk of bias. The use of this scale is appropriate because the randomization process of the trials did not apply to this review as only data from the ACDF trial arms were extracted.

### Data Abstraction

Two independent reviewers collected data into Excel spreadsheets. Demographics, type and study risk of bias, mean preoperative and postoperative VAS and/or NDI scores were recorded at multiple points in time (preoperatively, and postoperatively at 6 weeks, 3, 6, 12, 24, 36, and 48 months). Research ethics board approval is not required as it examines data from published studies. Therefore, there are no concerns regarding patient privacy or participant ethics.

### Statistical Analysis

Demographic data is pooled for age, gender, smoking status, and NDI and VAS scores. The pooled proportions and pooled mean with standard deviation are reported. Some studies utilized a 100-point VAS neck pain scale while others utilized the more traditional 10-point scale. These scores are documented in the tables as reported by authors; however, the 10-point scale was selected for formal analyses and the 100-point scores were therefore divided by 10 to standardize the pain score from 0 and 10. In order to evaluate the mean change in pain scores, the mean pain scores from the preoperative assessment and at each follow-up visit were pooled from 32 included studies. The pooled means were compared to preoperative pooled means at each follow-up visit by use of a random-effects model with inverse variance. Between-study heterogeneity was tested using χ^2^ test and quantified by *I*^2^ statistics. The overall pooled mean change in pain scores with 95% confidence intervals (CIs) over 24 months and heterogeneity are reported. Comprehensive Meta-Analysis version 3.3.070 (Biostat, Inc, Englewood, NJ) was used for meta-analysis.

## Results

**Figure 1.**
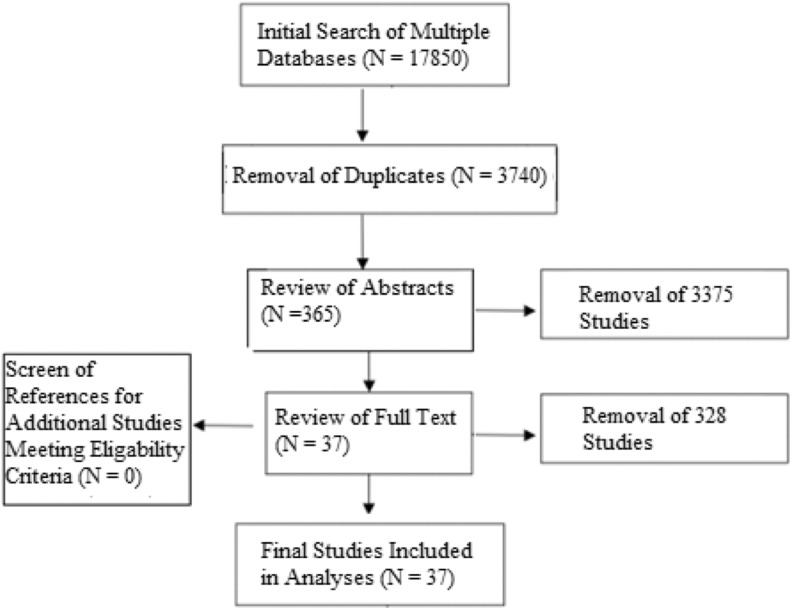
Flow diagram of search strategy with included studies.

Initial librarian-assisted search revealed 17 850 studies, which after deletion of duplicates and initial title screen for relevance left 3740 studies for abstract review (Figure 1). Following abstract review, 3375 studies were removed for failure to meet eligibility criteria. The remaining 365 studies were examined via full text review. Discrepancies were resolved between authors and 37 studies were included in final analyses.^4-40^ Individual studies are presented in Table 1. Detailed information regarding studies and collected data is available in the appendix, available online.

**Table 1.** A List of Studies Included in the Analyses.

Study	Year	VAS	NDI	Study	Year	VAS	NDI
Zoëga et al Group 1^4^	1998	X		Maldonado et al^22^	2011	X	X
Zoëga et al Group 2^4^	1998	X		Zhang et al^23^	2012	X	X
Porchet et al 20 04^5^	2004	X	X	Coric et al^24^	2013	X	X
Chen et al^6^	2005	X		Ha et al Group 1^25^	2013	X	X
Schils et al Group 1^7^	2006	X		Ha et al Group 2^25^	2013	X	X
Schils et al Group 2^7^	2006	X		Chen et al^26^	2013	X	X
Bindal et al^8^	2007		X	Kasliwal et al Group 1^27^	2013	X	X
Mummaneni et al^9^	2007		X	Kasliwal et al Group 2^27^	2013	X	X
Nabhan et al^10^	2007	X		Kasliwal et al Group 3^27^	2013	X	X
Oktenoglu et al^11^	2007	X		Phillips et al^28^	2013	X	X
Cosar et al^12^	2008	X		Zigler et al^29^	2013	X	X
Fernández-Fairen et al Group 1^13^	2008	X	X	Janssen et al^30^	2015	X	
Fernández-Fairen et al Group 2^13^	2008	X	X	Skeppholm et al^31^	2015	X	X
Bhadra et al Group 1^14^	2009	X		Arnold et al Group 1^32^	2016	X	X
Bhadra et al Group 2^14^	2009	X		Arnold et al Group 2^32^	2016	X	X
Bhadra et al Group 3^14^	2009	X		Hisey et al^33^	2016	X	X
Heller et al^15^	2009		X	Loumeau et al^34^	2016	X	X
Murrey et al^16^	2009		X	Richter et al^35^	2016	X	
Nabhan et al Group 1^17^	2009	X	X	Burkus et al^36^	2017		
Nabhan et al Group 2^17^	2009	X	X	Pandey et al^37^	2017	X	X
Burkus et al^18^	2010	X	X	Razankovic et al^38^	2017	X	
Delamarter et al^19^	2010	X	X	Sasso et al 201 7^39^	2017	X	X
Garrido et al^20^	2010	X	X	Arts et al Group 1^40^	2017		X
Löfgren et al Group 1^21^	2010	X	X	Arts et al Group 2^40^	2017		X
Löfgren et al Group 2^21^	2010	X	X				

Abbreviations: VAS, Visual Analogue Scale; NDI, Neck Disability Index.

Studies range in year from 1998 to 2017. A total of 2138 patients are analyzed with regard to VAS scores and 2477 patients with regard to NDI scores. Fifty-three percent of participants were male. Average time to final follow-up ranged from 6 months to 120 months for VAS (mean 33.1 ± 23.9 months) and NDI data (mean 34.5 ± 23.8 months).

### Risk of Bias

Each of the 37 studies underwent risk of bias by MINORS scale. The mean MINORS score was 19.9 (±2.2) and ranged from 13 to 23 indicating that the risk of bias was overall acceptable for most of the included studies. Table 2 presents each study along with its MINORS score.

**Table 2.** A List of Studies Included Along With Respective MINORS Scores for Quality Assessment.

Study	MINORS Score	Study	MINORS Score
Zoëga et al^4^	15	Zhang et al^23^	20
Porchet et al^5^	20	Coric et al^24^	22
Chen et al^6^	21	Ha et al^25^	21
Schils et al^7^	19	Chen et al^26^	20
Bindal et al^8^	14	Kasliwal et al^27^	22
Mummaneni et al^9^	21	Phillips et al^28^	21
Nabhan et al^10^	22	Zigler et al^29^	22
Oktenoglu et al^11^	13	Janssen et al^30^	21
Cosar et al^12^	19	Skeppholm et al^31^	20
Fernández-Fairen et al^13^	22	Arnold et al^32^	22
Bhadra et al^14^	18	Hisey et al^33^	18
Heller et al^15^	20	Loumeau et al^34^	20
Murrey et al^16^	20	Richter et al^35^	20
Nabhan et al^17^	20	Burkus et al^36^	19
Burkus et al^18^	22	Pandey et al^37^	18
Delamarter et al^19^	23	Razankovic et al^38^	20
Garrido et al^20^	21	Sasso et al^39^	19
Löfgren et al^21^	23	Arts et al^40^	18
Maldonado et al^22^	21	Mean ± SD	19.9 ± 2.2

Abbreviation: MINORS, Methodological Index for Non-Randomized Studies.

### Axial Neck Pain

**Figure 2.**
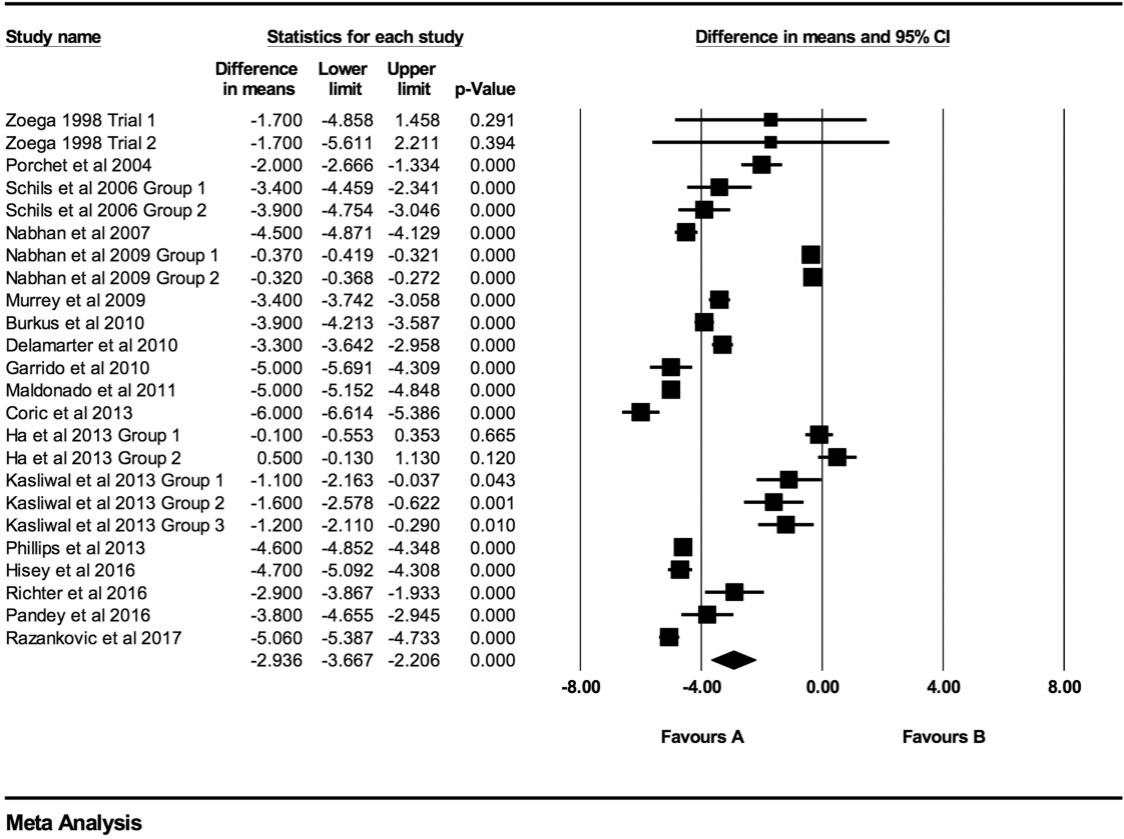
VAS baseline scores compared to 3-month follow-up (−2.94 [95% CI: −3.67 to −2.21]; heterogeneity: *I*^2^ = 99.7%, *P* < .001).

**Figure 3.**
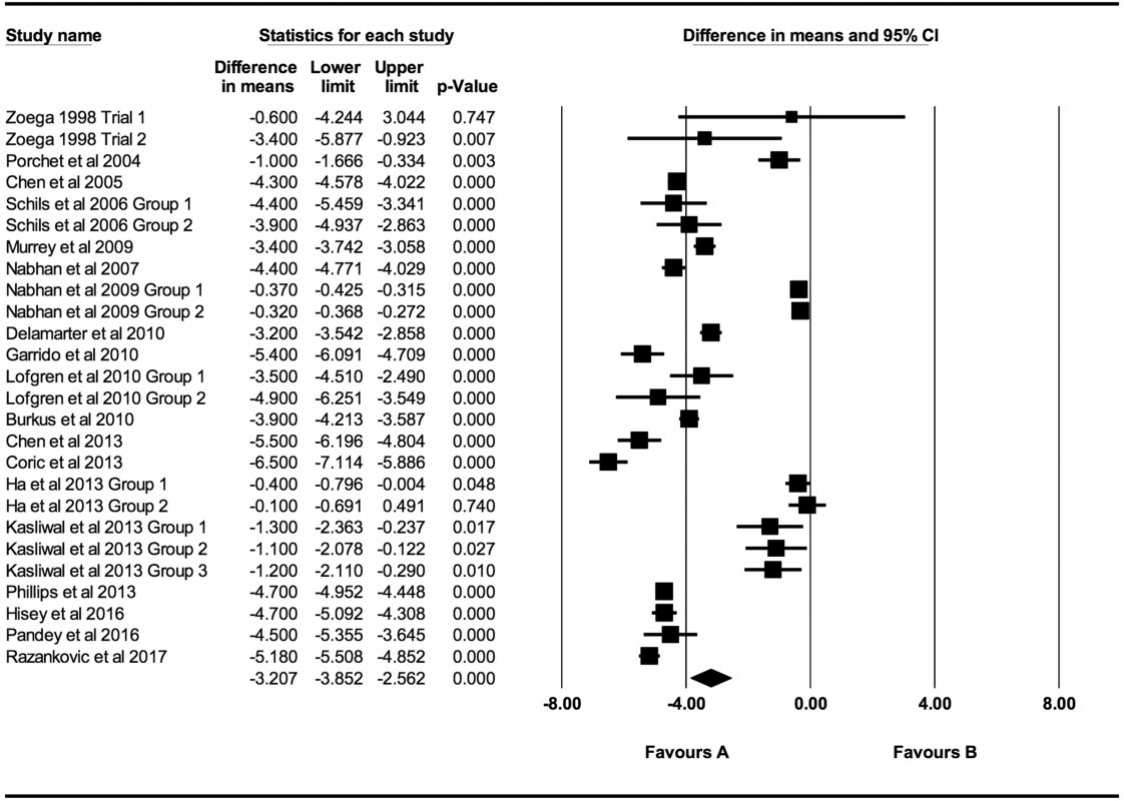
VAS baseline scores compared to 6-month follow-up (−3.21 [95% CI: −3.85 to −2.56]; heterogeneity: *I*^2^ = 99.5%, *P* < .001).

**Figure 4.**
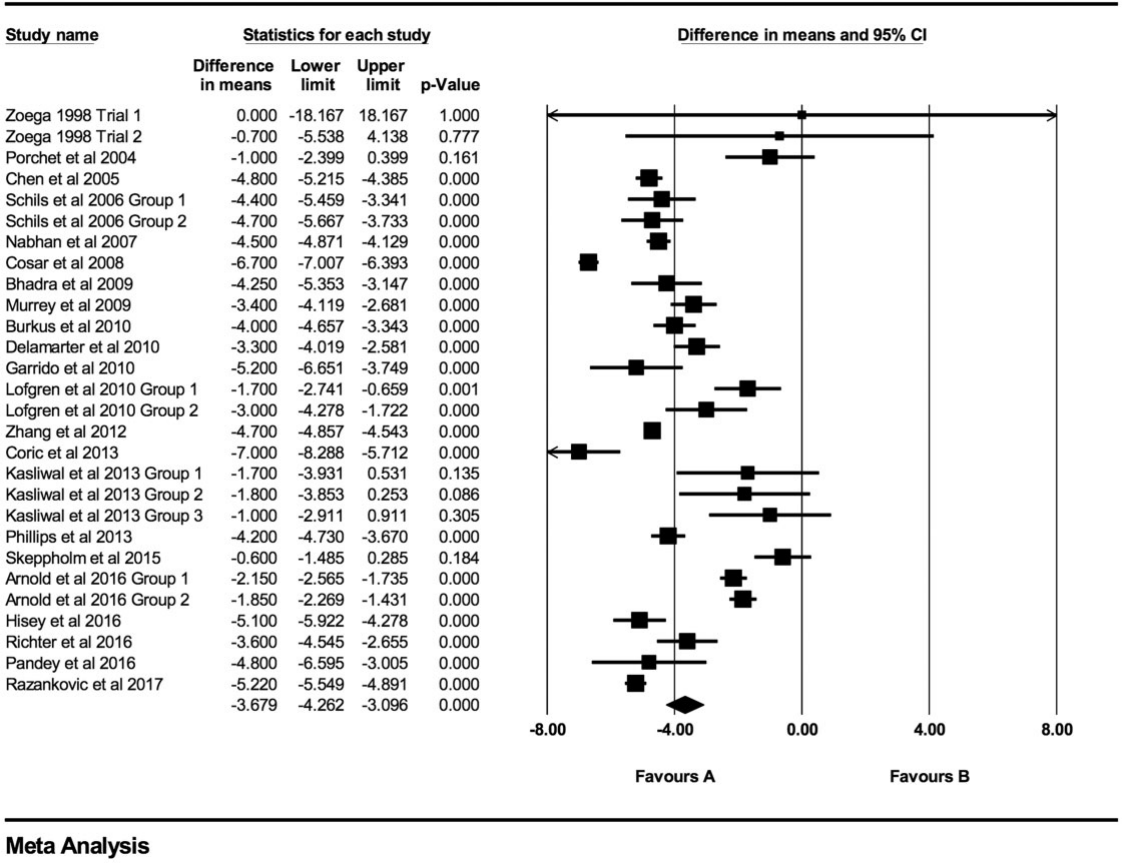
VAS baseline scores compared to 12-month follow-up (−3.68 [95% CI: −4.26 to −3.10]; heterogeneity: *I*^2^ = 98.1%, *P* < .001).

**Figure 5.**
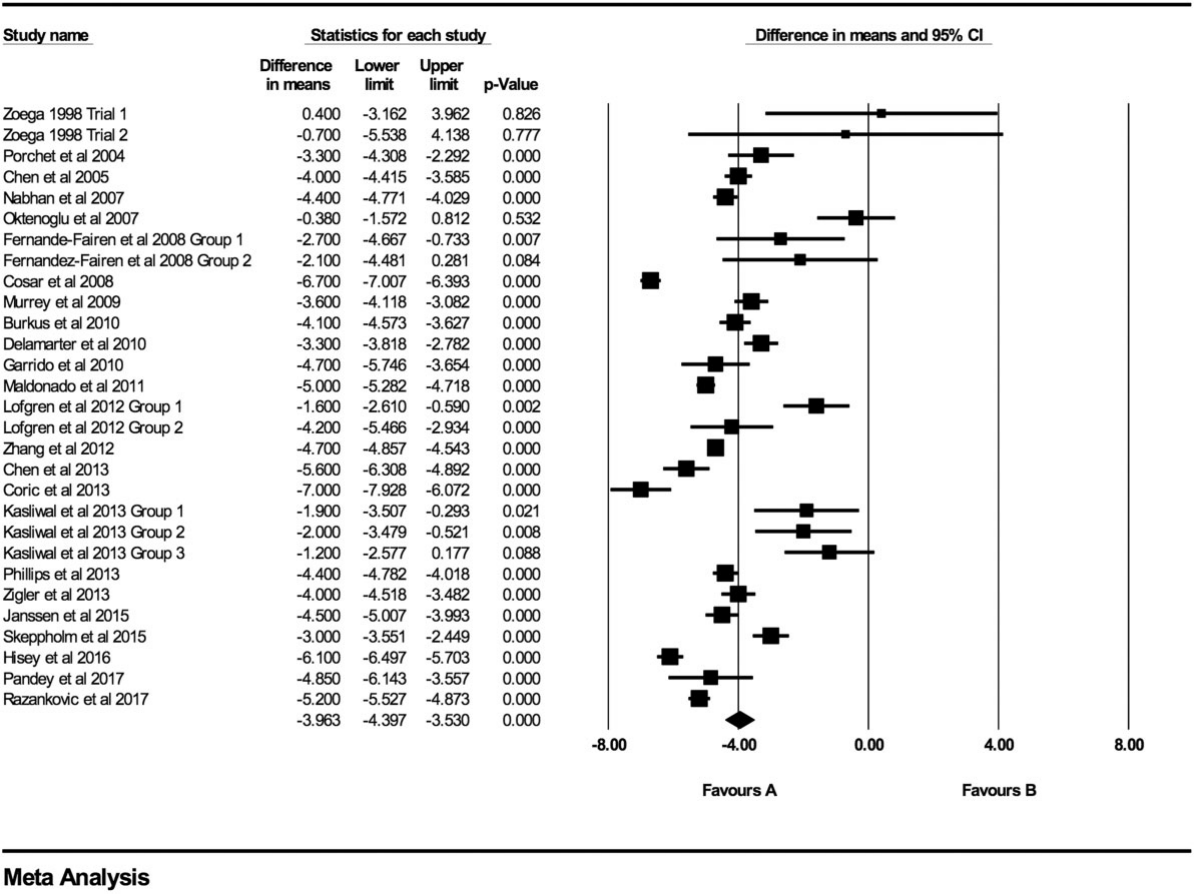
VAS baseline scores compared to 24-month follow-up (−3.96 [95% CI: −4.40 to −3.53]; heterogeneity: *I*^2^ = 94.7%, *P* < .001).

**Figure 6.**
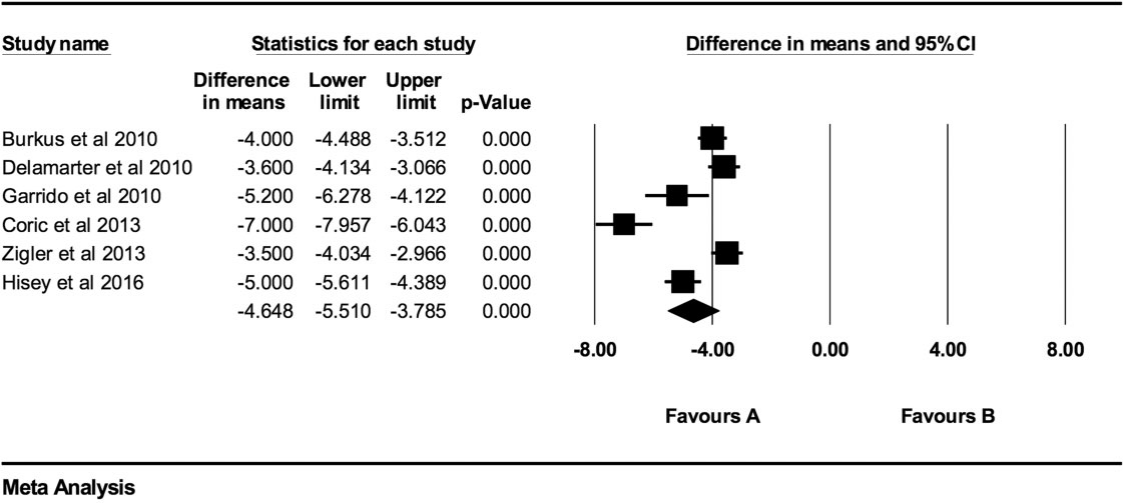
VAS baseline scores compared to 48-month follow-up (−4.65 [95% CI: −5.51 to −3.79]; heterogeneity: *I*^2^ = 90.9%, *P* < .001).

At every time point there was a statistically significant decrease in both VAS and NDI scores. Individual VAS mean differences are reduced compared postoperative scores at 6 weeks, 3 months, 6 months, 12 months, 24 months, 48 months, and >48 months follow-up were −2.52 (95% CI: −3.46 to −1.59; *P* < .0001; *I*^2^ = 99.9%; Figure A1), −2.94 (95% CI: −3.67 to −2.21; *P* < .0001; *I*^2^ = 99.7%; Figure 2), −3.21 (95% CI: −3.85 to −2.56; *P* < .0001; *I*^2^ = 99.5%; Figure 3), −3.68 (95% CI: −4.26 to −3.10; *P* < .0001; *I*^2^ = 98%; Figure 4), −3.96 (95% CI: −4.40 to −3.53; *P* < .0001; 94.7%; Figure 5), −4.65 (95% CI: −5.51 to −3.79; *P* < .0001; 90.9%; Figure 6), −4.71 (95% CI: −5.83 to −3.58; *P* < .0001; *I*^2^ = 93.0%; Figure A2), respectively.

**Figure 7.**
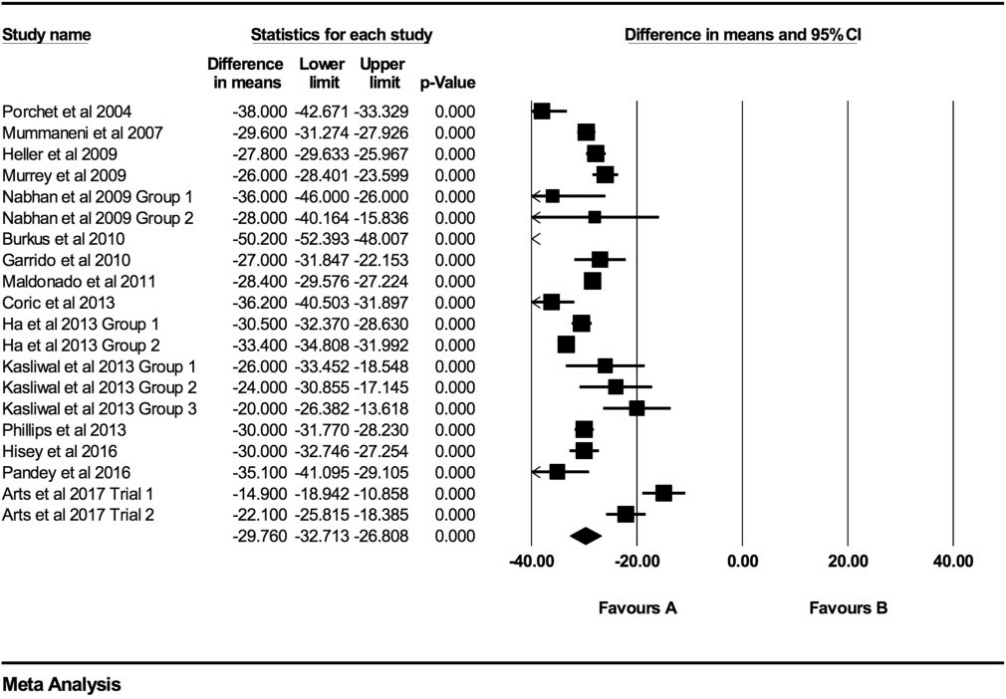
NDI baseline scores compared to 3-month follow-up (−29.76 [95% CI: −32.71 to −26.81]; heterogeneity: *I*^2^ = 96.0%, *P* < .001).

**Figure 8.**
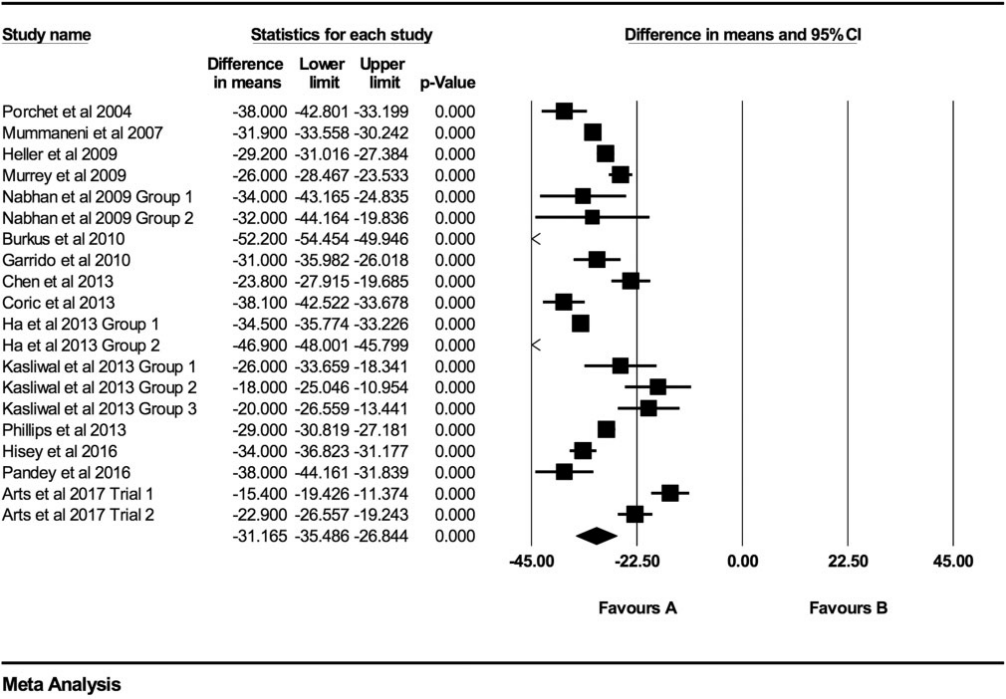
NDI baseline scores compared to 6-month follow-up (−31.17 [95% CI: −35.49 to −26.84]; heterogeneity: *I*^2^ = 98.1%, *P* < .001).

**Figure 9.**
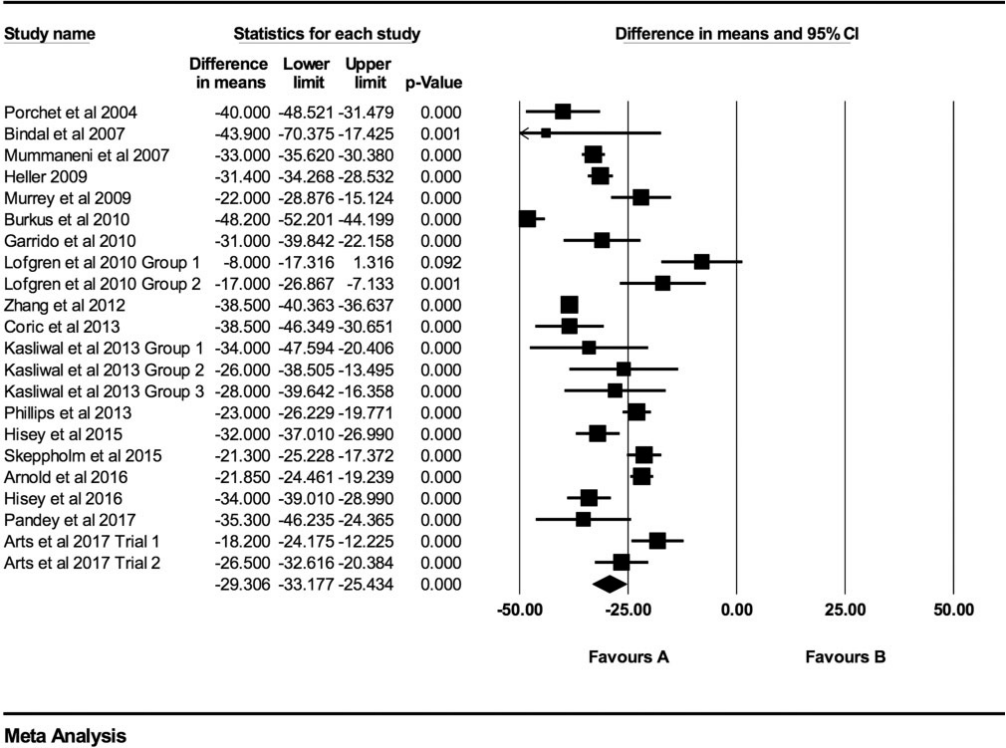
NDI baseline scores compared to 12-month follow-up (−29.31 [95% CI: −33.18 to −25.43]; heterogeneity: *I*^2^ = 93.0%, *P* < .001).

**Figure 10.**
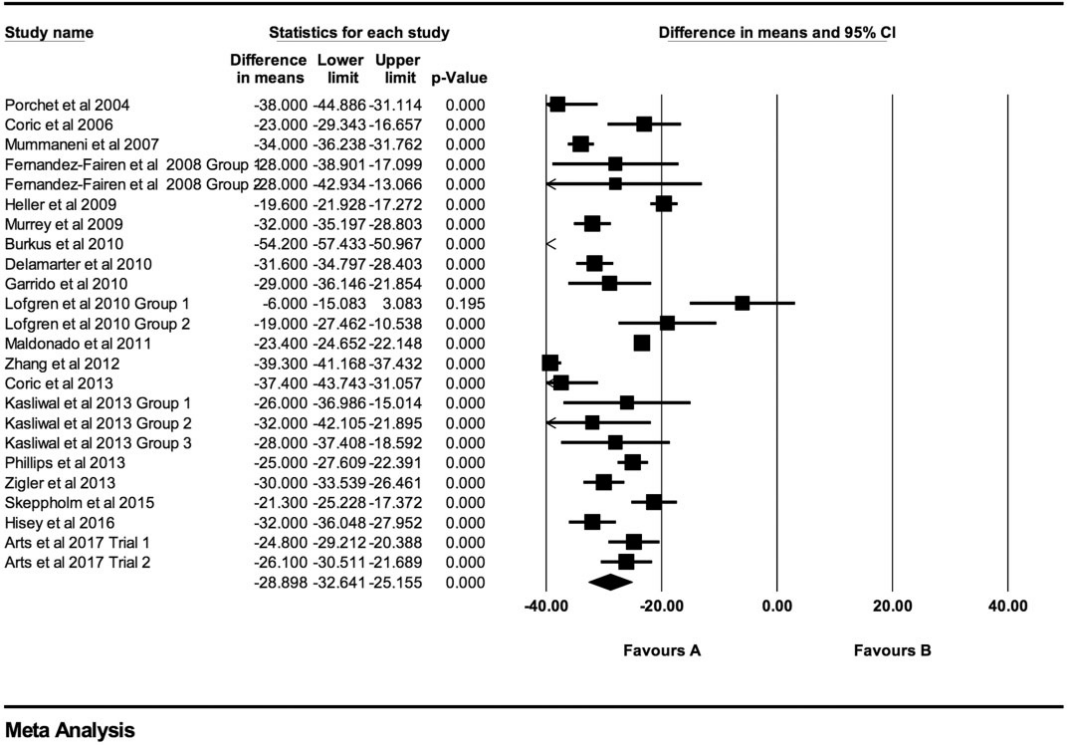
NDI baseline scores compared to 24-month follow-up (−28.90 [95% CI: −32.64 to −25.16]; heterogeneity: *I*^2^ = 96.12%, *P* < .001).

**Figure 11.**
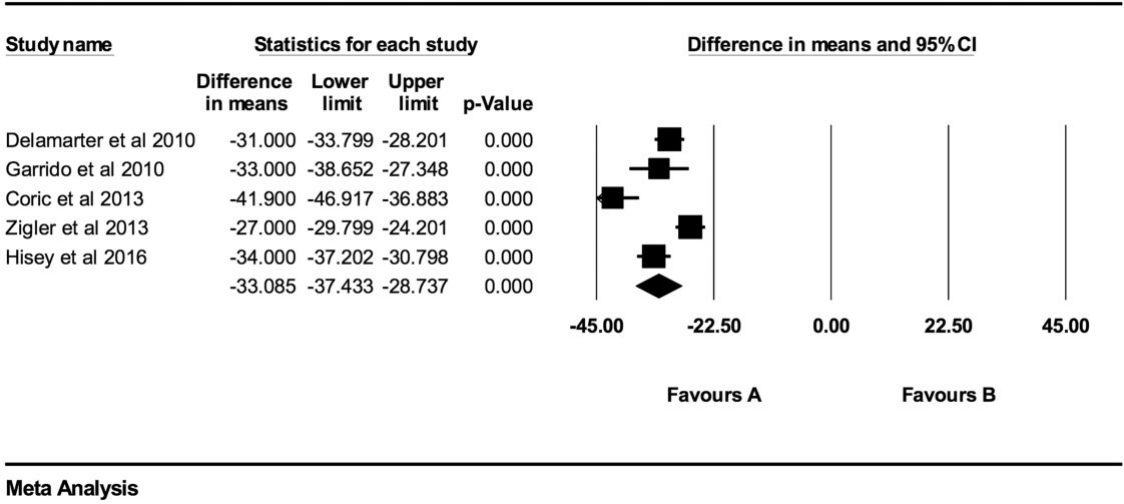
NDI baseline scores compared to 48-month follow-up (−33.09 [95% CI: −37.43 to −28.74]; heterogeneity: *I*^2^ = 86.3%, *P* < .001).

Individual NDI mean differences at 6 weeks, 3 months, 6 months, 12 months, 24 months, 48 months, and >48 months follow-up were −26.73 (95% CI: −30.88 to −22.59; *P* < .0001; *I*^2^ = 97.0%; Figure A3), −29.76 (95% CI: −32.71 to −26.81; *P* < .0001; *I*^2^ = 96.0%; Figure 7), −31.17 (95% CI: −35.49 to −26.84; *P* < .0001; *I*^2^ = 98.1%; Figure 8), −29.31 (95% CI: −33.18 to −25.43; *P* < .0001; *I*^2^ = 93.0%; Figure 9), −28.90 (95% CI: −32.64 to −25.16; *P* < .0001; *I*^2^ = 96.1%; Figure 10), −33.09 (95% CI: −37.43 to −28.74; *P* < .0001; *I*^2^ = 86.3%; Figure 11), and −37.61 (95% CI: −45.88 to −29.34; *P* < .0001; *I*^2^ = 91.0%; Figure A4), respectively.

## Discussion

Axial neck pain is a multifactorial problem associated with muscular, ligamentous, discogenic, and degenerative bony spinal anatomy. This meta-analysis demonstrates that in patients with myelopathy or radiculopathy resulting from single-level disc disease, there are consistent reductions in axial neck pain following ACDF. It is likely that the disc itself is the main neck pain generator in these patients as pain is relieved following discectomy and fusion, and motion-preserving disc arthroplasty. For ACDF, the effect is stable over time with similar mean reductions in VAS and NDI at all time points postoperatively. These results will help guide preoperative discussions with patients, and may accurately address patient expectations.

Minimally important differences (MIDs) are the smallest effects that a patient would consider beneficial to justify a procedure^41^ and have been studied for multiple different scales within the field of spinal surgery. The MID for the VAS is found to be 2.5 points for extremity,^42^ and 3.5 points for VAS axial pain.^42^ The MID for NDI is determined to be 7.5 points.^43^ While the NDI data suggest a clinically significant reduction in axial neck pain at all time points, the VAS data presented here exceed the MID 12 months following ACDF. Our study finds a 28.8-point decrease in NDI at 24 months as well as a 4.1-point decrease in VAS for neck pain. The precise estimates and large number of included studies suggest a comprehensive evaluation of the current evidence of ACDF on the outcome of neck pain.

While this study reports clinically meaningful and statistically significant reductions in neck pain following ACDF, we do not recommend ACDF for neck pain in the absence of currently accepted indications for surgery. Selection bias is present as included patients were enrolled due to the presence of cervical myelopathy or radiculopathy. Accordingly, interpreting a causal relationship between ACDF and neck pain in the absence of neural compression warrants caution. One group of surgeons from Washington University report a systematic review on 3 case series utilizing ACDF for the purpose of mechanical/axial neck pain in patients without radiculopathy or any other standard indications for the procedure. They were criticized by editorial staff for presenting limited data to produce a meaningful analysis; however, they are commended for venturing beyond the accepted standards for this procedure. The 3 studies they reported^44-46^ included a total of 166 patients showing a 50% to 60% decrease in axial neck pain at 4-year follow-up and patient satisfaction rates ranging from 56% to 79%.^3^ Functional outcomes were improved between 32% and 52% from baseline.^3^

Future research may attempt to understand the direct relationship between ACDF and axial neck pain in patients with single-level disc disease. It is unethical to randomize patients to surgical treatment for neck pain, as ACDF is not accepted as a treatment for this condition. However, the current treatment guidelines for myelopathy suggest that mild myelopathy may be treated conservatively or surgically. Therefore, clinical equipoise exists to design a study examining patients with mild myelopathy, with and without axial neck pain, to determine the treatment effects of ACDF on axial neck pain directly. The degree to which axial neck pain is affected by other treatments for myelopathy (eg, laminectomy, laminoplasty, anterior corpectomy, and fusion) remains to be determined. It would be useful for future research to fully characterize the effects of these procedures on axial neck pain.

There are multiple strengths in this meta-analysis. A thorough search strategy is used with various keywords and a comprehensive search of multiple databases is undertaken. There are multiple reviewers involved in the process of article retrieval, risk of bias assessment, and data abstraction to ensure accuracy and minimize bias. The included studies are multicenter trials, including multiple implant products and surgeons, which increases the generalizability of our results. While we demonstrate high levels of heterogeneity in our outcomes at several time points, a large majority of studies were in favor for ACDF for a reduction in axial neck pain. Accordingly, we do not consider this inconsistency in treatment effect to weaken our study’s conclusion.

## Conclusions

Our results indicate that ACDF is associated with a significant reduction in axial neck pain compared to preoperative values in patients being treated for myelopathy or radiculopathy. These effects meet clinically significant MIDs and may influence patients’ expectations for surgery. These results may help clarify expected surgical outcomes in preoperative discussions.

## Supplemental Material

ACDF_Supplementary_Files - The Role of Anterior Cervical Discectomy and Fusion on Relieving Axial Neck Pain in Patients With Single-Level Disease: A Systematic Review and Meta-Analysis Click here for additional data file. ACDF_Supplementary_Files for The Role of Anterior Cervical Discectomy and Fusion on Relieving Axial Neck Pain in Patients With Single-Level Disease: A Systematic Review and Meta-Analysis by Colby Oitment, Tracy Watson, Victor Lam, Mohammed Aref, Alex Koziarz, Edward Kachur, Jetan H. Badhiwala, Saleh A. Almenawer and Aleksa Cenic in Global Spine Journal
