# Supplementary material for: The Role of Anterior Cervical Discectomy and Fusion on Relieving Axial Neck Pain in Patients With Single-Level Disease: A Systematic Review and Meta-Analysis
Source: Global Spine J. 2019 Mar 25;10(3):312–23. doi: 10.1177/2192568219837923 (PMC7160803; doi:10.1177/2192568219837923)
Supplement: ACDF_Supplementary_Files - The Role of Anterior Cervical Discectomy and Fusion on Relieving Axial Neck Pain in Patients With Single-Level Disease: A Systematic Review and Meta-Analysis [file ACDF_Supplementary_Files.pdf]

APPENDIX

Table A1: Demographic information of included studies

Study	Level of evidence	Study Characteristics	Outcomes
Zoega et al 1998	Level 2	A randomized control trial conducted at Sahlgrenska University Hospital in Sweden. This is a full article written in English. Total number of participants: 27; Female: 12; mean age 41 (range 25 – 60); Stabilization with graft and cervical spine locking plate: 15 (Female: 8); Grafting without fixation: 12 (Female: 4); smoking status not recorded; BMI not recorded.	VAS for neck pain with plate group: preop = 5.4 (range – 3.1 – 8.8), 24 months = 5.8 (range = 1.7 – 8.8); VAS for neck pain without plate: preop = 6.3 (range = 4.4 – 9.1); 24 months = 5.6 (0-8.2)
Porchet et al 2004	Level 1	A randomized control trial, patient location unspecified, author affiliated with Hospital in Switerland. This is a full article written in English. Total number of participants: 55; Female: 26; ACDA + prestige II disc: 27; ACDF + iliac crest autograft: 28 (Female: 16; mean age = 43± 6.9; smoking: 11); BMI not recorded	For ACDF group NDI pre: 80; NDI 24 months post 22; VAS pre = 14.8; VAS post = 5.5. At 2 years 46 patients in total were lost to follow up from both groups.
Chen et al 2005	Level 3	A prospective cohort trial conducted at Chang Gung Memorial Hospital at Taiwan. This is a full article written in English. The number of participants: 63; Female: 29; mean age: 50.4 (range = 27 – 64) All patients received	All surgical procedures were technically successful, and there were no complications related to anesthesia or the overall procedure. There was no case of PMMA cage dislodgment nor cage failure. The fusion rates

		underwent anterior cervical microdiscectomy and implantation of a PMMA cervical cage filled with autologous cancellous bone; smoking: 12; BMI not recorded	were 90.5 and 100% at the 6- and 12-month follow-up examinations. Based on the Huskisson VAS scoring, neck pain had decreased from 71 ± 13 mm preoperatively to 28 ± 17 at 6, 23 ± 19 at 12, and 31 ± 19 mm at 24 months
Schils et al 2006	Level 3	A prospective cohort trial conducted at University Hospital of Geneva in Switzerland. The is a full article written in English. The number of participants: 36; Female: 14. In all patients conservative treatment had failed to resolve symptoms. Anterior cervical discectomy (ACD) + empty carbon fiber composite frame cage (CFCFC): 24 (Female: 10; mean age = 49); ACD + iliac crest autograft: 12 (Female: 4; mean age: 43); Patients with evidence of cervical instability, so-called whiplash syndrome, systemic infection, metabolic bone disease, active malignancy; previous cervical disc surgery, bilateral or multilevel symptomatic radicular compression, myelopathy, or psychiatric disease were excluded; smoking status not recorded; BMI not recorded	In cage-treated patients, neck pain preoperative VAS score: 6.4 ± 3.0; postoperative VAS score: 2.0 ± 2.0 at 12 months. In autograft-treated patients, neck pain VAS preoperative: 7.2 ± 1.6; VAS at 12 months: 2.5 ± 1.8.
Bindal et al 2007	Level 3	A prospective cohort trial conducted at Houston, Texas in the United States. This is a full article written in English. The number of	ACDF + Mystique instrumentation NDI preoperatively: 64.0 (range: 34-88); NDI 1 year follow up: 20.1 (range: not reported)

		participants: 24. ACDF + instrumentation using a resorbable Mystique anterior cervical plate: 24; Female: 12; mean age: 47.2±9.3 Smoking: 9; BMI status not recorded	
Mummaneni et al 2007	Level 1	A randomized control trial with data collected from 32 investigation sites with the United Staes. This is a full article written in English. The number of participants: 541; anterior cervical discectomy and decompression and arthroplasty with the PRESTIGE ST Cervical Disc System (Medtronic Sofamor Danek): 276 (Femael: 156); ACDF: 265 (Female: 143; smoking: 92; mean age: 43.9); BMI status not recorded	ACDF group follow up at 24 months: 75%; ACDF preop NDI: 56.4; ACDF 24 month postop NDI: 22.4
Nabhan et al 2007	Level 2	A randomized control trial conducted at Homburg, Germany. This is a full article written in English. The number of participants: 33; Mean age of all participants: 45±11; Female: 14; Disc replacement: 16; ACDF: 17; BMI and smoking status not recorded	VAS score for ACDF group preoperatively: 6.4±0.9; 24 weeks post op: 2.0±0.5
Oktenoglu et al 2007	Level 2	A randomized control trial conducted in Turkey. The is a full article written in English. The number of participants: 20; Group A consisted of 11 patients (4 men and 7 women; median age 39.9 years of age) who underwent simple ACD. Group B was comprised of 9	For ACDF group, the VAS score preoperatively: 3.22 and 1-year postoperative VAS: 2.0

		patients (7 men and 2 women; median age 40.2 years of age) ACDF. BMI and smoking status were not recorded.	
Cosar et al 2008	Level 3	A prospective cohort trial with authors affiliated from several institutions including Turkey and United States. Number of participants: 17; Female: 9; Mean age: 46 (range: 33 to 60 y); mean BMI 25.3 (range: 20.1 to 31.2); Smoking status: 7. All patients had ACDF using tricalcium phosphate and hydroxyapatite ([beta]-TCP/HA) grafts.	VAS score for neck pain was 8.1$\pm$0.73 preoperatively and 1.4$\pm$0.51 postoperatively at 18 – 24 months.
Fernández-Fairen et al 2008	Level 2	A randomized control trial with authors from Spain. Number of participants: 61; 28 patients received interbody porous tantalum implant (Group 1) and 33 received interbody autologous bone graft plus anterior plate fixation; Group 1 consisted of 18 women and 10 men with a mean age of 47.5 years (range, 27–62), Group 2 was comprised of 21 women and 12 men with a mean age of 49.3 years (range, 22–65). Smoking status and BMI not recorded.	Pain on VAS 0 – 10 score before surgery: Group 1: 6.8 (range = 5-8); Group 2: 4.1 (range 5- 9); VAS score 24 months after treatment: Group 1: 4.1 (range 4-7); Group 2: 4.7 (range 2-8)/<br><br>NDI score preoperatively. Group 1: 46.8% (range 38 – 56); Group 2: 48.9# (range 32-66). NDI 24 months after surgery: Group 1: 19% (range 10 – 34); Group 2: 20.9 (10-40)
Bhadra et al 2009	Level 3	Sixty consecutive patients (15 each group), mean age 36 (range 24–76 years) with single-level cervical disc disease underwent surgical treatment with four different techniques in two	The clinical outcome in terms of<br><br>VAS of neck and arm pain and SF12 physical and mental score improvement (P = 0.001)

		centers over the period of 1999–2005. The four groups were (1) plate and tricortical autograft, (2) plate, cage, and bone substitute, (3) cage only, and (4) disc arthroplasty. The data was collected prospectively according to our protocol and subsequently analyzed. The clinical outcome was assessed comparing visual analog scale (VAS) of neck pain and, short form 12 (SF12) questionnaire both pre- and postoperatively. The radiological assessment was done for fusion rate and postoperative related possible complications at 3 months, 6 months, 1 year, and final follow-up. The cost analysis was<br><br>done calculating the operative time, hospital stay, implant cost together. The mean follow-up period was 31 months (range 28–43 months).	were comparable with all<br><br>four techniques. The radiological fusion rate was comparable to current available data. As the hospital stay was<br><br>longer (average 5 days) with plate and autograft group, the total cost was maximum (average £2,920) with this group. There was satisfactory clinical and radiological outcome with all four techniques. Using the cage alone was the most cost-effective technique, but the disc arthroplasty was comparable to the use of cage and plate.
Heller et al 2009	Level 1	A prospective, randomized, multicenter study of surgical treatment of cervical disc disease with 24 months follow up. 242 underwent Bryan cervical disc arthroplasty and 221underwent a single-level anterior cervical discectomy and decompression and fusion. Main outcome was neck disability index (NDI) and was analyzed at 12 and 24 months. Smoking status and BMI not recorded.	Neck disability index was the main outcome and it showed improvement at 12 and 24 months however at 24 months the artificial disc group had statistically greater improvement. Rate of adverse events was 1.7% in investigational group and 3.2% in control group.

Murrey et al 2009	Level 1	This is a randomized controlled clinical trial comparing outcomes in patients who underwent ProDisc-C vs ACDF. 87 patients included in this study which comprises two sites of the 13 centers that were included. Outcomes were assessed using NDI, VAS and SQ-36.	both groups showed statistically significant improvement over their preoperative baseline with regard to NDI, VAS arm and neck pain levels, SF-36 mental composite score (MCS), and physical composite score (PCS) (*P* < .05). At 24 months, the disc replacement group showed results equivalent to the ACDF group with regard to NDI, VAS arm and neck pain, and SF-36 MCS. At 24 months, the disc replacement group showed significantly greater improvement in SF-36 PCS as compared to the ACDF group (*P* = .0359). Of note, there was a trend toward greater patient satisfaction in the discreplacement group as compared to the ACDF group (83% versus 71%, *P* = .144)
Nabhan et al 2009	Level 2	This is is prospective randomized and controlled trial to compare outcomes in bone density, clinical outcomes, MRI compatability, and change in bone density of a cervical spine that was treated with either bioabsorbable or titanium plates. 40 patients with single level cervical radiculopathy were randomized to one of the two groups. For follow up the NDI and VAS was used to assess neck pain. Radiostereometry was performed immediately postoperative and after 6 weeks, 3, and 6	Three-dimensional analysis of segmental motion (medio-lateral, cranio-caudal and anterior-posterior) did not reveal any statistical difference between both groups at any time postoperatively (P>0.05). Fusion rate and speed evaluated on Radiostereometric analysis and computed tomography of cervical spine segment were similar in both groups. The VAS and NDI did not differ between both groups after 6 months (P>0.05).

		months. MRI of the cervical spine was obtained immediately postoperatively at 3 and 6 months to assess hematoma, infection, and swelling. Computed tomography of the operated cervical spine segment was performed to assess bone density, expressed in Hounsfield units.	
Burkus et al 2010	Level 2	Prospective, nonblinded study, 541 patients at 32 investigational sites were randomly assigned to 1 of 2 treatment groups. Group 1 underwent replacement with prestige disc prosthesis, and Group 2 underwent an instrumented interbody fusion. The results of the investigational group, in which patients received the Prestige disc prosthesis, were compared with those of the control group, in which patients underwent an instrumented interbody fusion. Data were collected preoperatively, intraoperatively, and at 1.5, 3, 6, 12, 24, 36, and 60 months postoperatively. To date, 271 patients have completed 5 years of clinical follow-up (144 investigational and 127 control patients).	improvements in Neck Disability Index (NDI) scores, Physical Component Summary scores<br><br>of the 36-Item Short-Form Health Survey, and neck and arm pain scores were achieved by 1.5 months in both groups and sustained at 5 years. The mean NDI improvements from preoperative scores were 35.4, 36.3, and 38.4 at 24, 36, and 60 months, respectively, in the investigational group. The corresponding mean NDI improvements were 33.9,31.3, and 34.1 in the control group. The intergroup differences at both 36 and 60 months were significant ($p = 0.008$ and 0.022, respectively). The overall rates of maintenance or improvement in neurological status in the investigational<br><br>group were 91.6%, 92.8%, and 95.0%, respectively, at 24, 36, and 60 months compared with 83.6%, 83.2%, and 88.9% in the control group ($p = 0.006$, 0.004, and 0.051,

			respectively). The implant effectively maintained angular motion, averaging more than 7.3° at 36 months and 6.5° at 60 months after surgery. No implant migration was observed up to 60 months. There were statistically significant differences between the investigational and control groups<br><br>with regard to the rate of revision and supplemental fixation surgical procedures performed subsequent to the index<br><br>procedure.
Delamarter et al	Level 1	This is a prospective randomized multicenter trial comparing outcomes in patients that underwent ACDF, vs ProDisc-C. Patients were evaluated at preoperatively, and postoperatively at 6 weeks and 3, 6, 12, 18, 24, 36, and 48 months. The NDI, and VAS were used to assess pain outcome. 103 patients were randomized to ProDISC-C and 106 to ACDF at 13 sites.	VAS pain and NDI score improvements from baseline were significant for all patients (*P* _ .0001) but did not differ among groups. VAS satisfaction was higher at all time points for<br><br>PDC-R versus ACDF patients (*P* _ .0499 at 48 months). The percentage of patients who responded yes to surgery again was 85.6% at 24 months and 88.9% at 48 months in the PDC-R group, 80.9% at 24 months and 81.0% at 48 months in the ACDF group, and 86.3% at 24 months in the CA group. Five PDC-R patients (48 months) and no CA patients (24 months) had index-level bridging bone. By 48 months, approximately 4-fold more ACDF patients required secondary surgery (3 of 103

			PDC-R patients [2.9%] vs 12 of 106 ACDF patients [11.3%], *P* _ .0292). Of these, 6 ACDF patients (5.6%) required procedures at adjacent levels. Three CA patients required secondary procedures (24 months).
Garrido et al 2010	Level 2	Prospective, randomized, controlled. Level 1 Evidence. A total of 47 patients were enrolled at our site as part of an ongoing multicenter prospectively randomized study investigating ACDF versus Bryan cervical disc prosthesis. Functional outcomes are now reported at 48 months follow-up for our cohort of participants. Neck disability index score (NDI), VAS neck and arm and SF-36 both physical and mental as well as complications and reoperations will be reported.	Functional outcome data collected at routine follow-up for 48-months has favorably demonstrated improved functional<br><br>outcomes for NDI, neck/arm pain VAS scores, and the SF-36 physical/mental health component scores for the Bryan arthroplasty<br><br>and ACDF cohorts. The NDI scores for the Bryan arthroplasty preoperatively was 51 and at 48 months 10. For ACDF preoperative NDI score was also 51 and at 48 months<br><br>16.7. At 48 months NDI success, measured by Z15 points NDI improvement demonstrated a 93.3% success for Bryan arthroplasty<br><br>and an 82.4%success for ACDF. VAS neck pain scores for the Bryan arthroplasty preoperatively was 76.2 and at 48 months<br><br>was 13.6. VAS neck pain scores for ACDF preoperatively was 80.6 and at 48 months was 28.1. Arm Pain scores were also<br><br>measured and for the Bryan arthroplasty

			preoperatively measured 78.8 and at 48 months 10.8. For ACDF arm pain scores<br><br>preoperatively measured 77.1 and at 48 months 21.7. During 48 months of follow up 6 had re-do surgeries in the control group and only 1 in the Bryan disc group.
Lofgren et al 2010	Level 2	A prospective, randomized, controlled study was carried out to compare the radiological and clinical outcomes after anterior cervical decompression and fusion (ACDF) with Trabecular MetalTM (TM) to the traditional Smith–Robinson (SR) procedure with autograft. 80 consecutive patients planned for ACDF were randomized for fusion with either TM or tricortical autograft from the iliac crest (SR) after discectomy and decompression. Digitized plain radiographic images of 78 (98%) patients were obtained preoperatively and at 2-year followup and were subsequently evaluated by two senior radiologists. Fusion/non-fusion was classified by visual evaluation of the A–P and lateral views in forced flexion/extension of the cervical spine and by measuring the mobility between the fused vertebrae. MRI of 20 TM cases at 2 years was successfully used to assess the decompression of the neural structures, but	Fusion rate in the SR group was 92%, and in the TM group 69% (P\0.05). The accuracy of the measurements was calculated to be 2.4_. Operating time was shorter for fusion with<br><br>TM compared with autograft; mean times were 100 min (SD 18) and 123 min (SD 23), respectively (P = 0.001).<br><br>The patients' global assessments of their neck and arm symptoms 2 years postoperatively for the TM group were<br><br>rated as 79% much better or better after fusion with TM and 75% using autograft. Pain scores and NDI scores were significantly improved in both groups when compared with baseline at all follow-ups, except for neck pain at 1 year for the TM group. There was no statistically significant difference<br><br>in clinical outcomes between fusion techniques or between patients who appeared

		was not helpful in determining fusion/non-fusion. Pain intensity in the neck, arms and pelvis/hip were rated by patients on a visual analog scale (VAS) and neck function was rated using the Neck Disability Index (NDI) the day before surgery and 4, 12and +4 months postoperatively. Follow-ups at 12 and 24 months were performed by an unbiased observer, when patients also assessed their global outcome.	radiologically fused or nonfused. There was no difference in pelvic/hip pain between patients operated on with or without autograft.
Maldonado et al 2011	Level 3	The objective of the study was to evaluate the incidence of adjacent segment disease in patients who underwent cervical disc arthroplasty (CDA) as compared with anterior cervical discectomy and fusion (ACDF). The study is a prospective cohort study of patients with a single-level cervical degenerative disc disease from C3 to C7 who underwent CDA or ACDF between January 2004 and December 2006, with a minimum follow-up of 3 years. The patients were evaluated pre- and postoperatively with the visual analog scale (VAS), the neck disability index (NDI), and a complete neurological examination. Plain radiographic assessments included sagittal-plane angulation, range of motion (ROM), and radiological signs of ASD.	One hundred and five patients underwent ACDF and 85 were treated with CDA. The postoperative VAS and<br><br>NDI were equivalent in both groups. The ROM was preserved in the CDA group but with a small decreased tendency<br><br>within the time. Radiographic evidence of ASD was found in 11 (10.5%) patients in the ACDF group and in 7(8.8%) subjects in the CDA group. The Kaplan–Meier survival analysis for the ASD occurrence did not reach statistically significant differences (log rank, P = 0.72).

Zhang et al 2012	Level 1	This is a prospective randomized controlled mulitcenter clinical trial comparing outcomes in patients who underwent arthroplasty vs. standard ACDF. A total of 120 patients from 3 large hospitals in China were randomly assigned to treatment with cervical disc arthroplasty (n = 60) using the BRYAN prosthesis or ACDF (n = 60) and were observed postoperatively for 24 months. The 2 groups had similar preoperative demographics and baseline characteristics including ROM, neck disability index, and visual analogue scale for neck and arm pain	The total disc replacement (TDR) group had a significantly longer operation time than the ACDF group ($P < 0.001$). Outcome data obtained after 24 months revealed a significant difference between the groups in<br><br>mean change from baseline in ROM at the index level ($P < 0.001$); ROM was maintained in the TDR group but reduced in the ACDF<br><br>group. There were no significant between-group differences in the baseline changes in neck disability index or visual analogue scale scores for pain. One patient in the TDR group and 4 patients in the ACDF group required reoperations.
Coric et al 2013	Level 2	Reports on results of 2 separate prospective randomized US FDA investigational divide exemption trials (Bryan Disc and Kineflex C) from a single site. Patients randomized to one of two arthroplasty options vs ACDF using structural allograft and anterior plating. Assessed at multiple time points (6 weeks, 3 months, 6 months, 12 months and yearly for 48 months). 74 patients enrolled (N = 33 ACDF), average follow up 6 years (86% patients follow up to 4 years.	Primary outcome NDI (61.3 baseline, 35.7 6 weeks, 25.1 3 months, 23.2 6 months, 22.8 12 months, 23.9 24 months for ACDF), VAS (8 baseline, 3 at 6 weeks, 2 at 3 months, 2 at 6 months 2 at 12 months 1.5 at 24 months 1 at 48 months), neurological examination (no differences). Blood loss, length of stay in hospital, complications (no differences).

		No differences in baseline demographic data between groups. Mean age 49.5 ACDF (No SD presented), Sex 16 males 25 females. No significant differences in blood loss, length of hospital stay. Remainder of demographic variables not presented (BMI, smoking).	
Ha et al 2013	Level 3	24 consecutive ACDF patients receiving ACDF with either low torque (group 1 n = 12) distraction or high torque distraction (group 2 n = 12) enrolled in study. Age and sex matched groups. 6 males/females per group. Mean age group 1 47.5 +/- 10.6 vs 48.1 +/- 8.4, operative level indicated by group. Operation time and intraoperative bleeding reported. No BMI or smoking status reported. Patients assessed on day 1,2,3 and 5, 1 month 3 months and 6 months postoperatively.	VAS for neck pain (0.8 +/- 1.1 at 1 month, 0.7 +/- 0/8 at 3 months, 0.4 +/- 0.5 at 6 months postoperatively in group 1 vs 0.4 +/- 1.3 +/- 1.1 at 1 month, 1.2 +/- 1.1 at 3 months and 0.6 +/- 0.7 at 6 months postoperatively in group 2).<br><br>NDI for neck pain (16.3 +/- 3.8 at 1 month, 13.8 +/- 3.0 at 3 months, 12.3 +/- 2.3 at 6 months postoperatively in group 1 vs 16.1 +/- 3.4 at 1 month, 14.1 +/- 2.7 at 3 months, 12.2 +/- 1.8 at 6 months postoperatively in group 2.<br><br>Disc height measurements taken.
Chen et al 2013	Level 2	Randomized prospective trial comparing TDR with Discover cervical disk arthroplasty vs ACDF using PEEK. N = 32 patients followed prospectively for single level symptomatic cervical disease (radiculopathy or myelopathy). All patients had cervical kyphosis less than 0 that spontaneously reduced on extension radiographs.	VAS scores (TDR 7.8 +/- 2.1 baseline vs 7.4 +/- 2.3 PEEK) compared to 6 months (2.3 +/- 1.0 TDR vs 1.9 +/- 0.9 PEEK) and 2 year scores TDR (2.2 +/- 0.8 vs PEEK 1.8 +/- 0.7)<br><br>NDI scores (preoperative 47.8 +/- 16.3 baseline TDR vs 45.2 +/- 13.7 PEEK) compared to 6 month (TDR 28.6 +/- 6.5 vs 21.4 +/- 7.4 in PEEK) and 2 year (TDR 16.5

		Baseline demographic data shows no differences between groups, Mean age TDR 43.2 +/- 10.2 vs ACDF 46.5 +/- 7.9. TDR 56.3% male vs ACDF 50% male. TDR smoking status 31.3% vss ACDF 25%. T2DM 12.5% in TDR vs 18.8% in ACDF.	+/- 6.2 vs 18.6 +/- 6.7 in PEEK.<br><br>Also assessed on Japanese Orthopedic Association JOA scores, clinical signs, activities of daily living (participant logs) and radiographically for signs of complication post-operatively.
Kasliwall et al 2013	Level 2	Prospective randomized non-blinded multicentre investigational device exemption study comparing porous tantalum ring (group 3 n = 13) device packed with autograft vs porous tantalum block (group 2 n = 15) device and iliac crest autograft control (group 1 n = 11). Conducted at 6 sites. Followed at 6 12 and 24 months post-operatively. Mean age in group 1 44.9 +/- 7.5 vs 44.6 +/- 11.2 in group 2 vs 46.4 +/- 9.9 in group 3. Weight presented in pounds and height separately for calculation of BMI. Group 1 36.4% male, vs group 2 53.9% male vs group 3 46.7% male. Marital status presented. Education level presented. Smoking status in group 1 54.6% vs group 2 46.2% vs group 3 53.3%. Alcohol use presented.<br><br>Operative time for 3 groups, blood loss, hospital stay and level of surgery presented separately.	NDI (presented in tables, Group 1 52 vs group 2 50 vs group 3 58 baseline with no standard deviations) compared to 6 weeks (group 1 34 vs group 2 28 vs group 3 38) vs 3 months (group 1 28 vs group 2 28 vs group 34) vs 6 months (group 119 vs group 2 31 vs group 3 29) vs 12 months (group 1 26 vs group 2 21 vs group 3 29) vs 24 month data (group 1 24 vs group 2 19 vs group 3 31), SF36, VAS for both neck and arm pain. Neck pain scores preoperatively (group 1 3.5 vs group 2 3.2 vs group 3 3.2) compared with 6 weeks (group 1 2.1 vs group 2 1.9 vs group 3 2.2) vs 3 months (group 1 2.4 vs group 2 1.9 vs group 3 2.2) vs 6 months (group 1 2.1 vs group 2 2.2 vs group 3 2.2) vs 12 months (group 1 1.9 vs group 2 1.4 vs group 3 2.1) vs 24 months (group 11.8 vs group 2 1.4 vs group 3 2.0). No standard deviations presented. Followed radiographically for signs of subsidence or non-union.

Phillips et al 2013	Level 1	Prospective multicenter randomized clinical trial comparing PCM cervical disc with ACDF using allograft and plate. Non-inferiority design. N=416 (N = 192 ACDF) at 24 sites across United States. N = 185 received ACDF after randomization. Mean age 45.3 +/- 9.0 years vs 43.7 +/- 8.3 in ACDF. 48.2% female vs 48.1% in ACDF. Reports race. BMI 28.2 vs 27.3 in ACDF. 51.8% smokers vs 48.6% smokers in ACDF. Reports workers compensation. Reports on myeloradiculopathy vs radiculopathy vs myelopathy for indications for surgery. Level operated, surgical time, blood loss and hospitalization days are reported by group.	NDI > 20% improvement (PCM 83.4% vs ACDF 81.5%) vs NDI > 15 point improvement (79.7% in PCM vs 75.5% in ACDF) vs Neck pain VAS (> 20 point improvement) 74.3% in PCM vs 75.5% in ACDF. Arm pain VAS reported. SF36 PCS and MCS for > 15% improvement reported. Neurological status (maintained or improved 94.7% in PCM vs 89.5% in ACDF. Dysphagia postoperatively and Nurick scale (maintained or improved) reported by group. Patient satisfaction reported and Odom criteria (poor-excellent) reported by group.<br><br>Radiographic analysis includes disc height, adjacent level degeneration, presence of heterotopic ossification,
Zigler et al 2013	Level 1	Randomized controlled trial comparing ProDisc-C and ACDF with 2 year follow-up. FDA regulated post-approval study. Patients evaluated at 6 weeks, 3 months, 6 months 12 and 18 months and annually for 5 years. N = 209 patients enrolled (N = 106 ACDF), Mean age 42.1 +/- 8.4 for TDR vs 43.5 +/- 7.1 ACDF. TDR 44.7% male vs ACDF 49% male. Smoking status 33% TDR vs 34.9% ACDF current smokers. Operative level by group is listed. Intraoperative time vs blood	Mean NDI neck and arm (Neck scores 53.93 TDR vs 52.28 ACDF preoperatively vs 22 TDR 22 ACDF at 2 year and 20 TDR vs 24 ACDF at 5 year), standard deviations not presented.<br>VAS neck and arm (Neck scores preoperatively TDR 72 vs ACDF 64 compared at 2 years TDR 28 vs 27 ACDF, 5 year data TDR 21 vs ACDF 30), no standard deviations presented.<br><br>Complications requiring revision surgery

		loss vs length of hospital stay is described.	presented separately for both groups.
Janssen et al 2015	Level 1	Randomized, controlled, multicenter trial comparing outcomes for Total Disc Arthroplasty (ProDisc-C) to ACDF. 209 patients recruited from August 2003-October 2004 and randomized to TDA (n=103) or ACDF (n=106). Baseline patient demographics: female (ACDF 54/106, TDA 55/103), BMI (ACDF 27.34±5.54, TDA 26.44±5.32), smoking status (ACDF 35/106, TDA 22/103).<br><br>Inclusion Criteria: radiculopathy, single level DDD between C3-7, failure of ≥6 weeks conservative management, NDI >15.<br><br>Primary outcomes: NDI, SF-36, neurologic success (defined in study), secondary surgical procedures, adverse events, VAS neck and arm pain scores, satisfaction scores, and radiographic analysis.<br><br>Assessments preoperatively and at 6 weeks, 3, 6, 12, 18, 24, 36, 48, 60, 72, 84 months postoperatively. 7 year follow-up 92%	All outcomes except SF-36 improved at 2- and 7-year follow up. Baseline NDI (ProDisc-C 53.9±15.1, vs ACDF 52.3±14.5), Baseline VAS neck pain (ProDisc-C 73.0±19.5, vs ACDF 65.7±21.7), Baseline VAS arm (ProDisc-C 63.9±28.8, vs ACDF 61±26.2). No significant difference between groups at 7 years in NDI<br><br>(p=0.1451), neurologic success (p=1.00), adverse events (p=0.8783), SF-36, VAS satisfaction (p=0.3906).<br><br>Significant difference between groups in range of motion at 7 years (ProDisc-C 8.12 ±5.91, ACDF 0.66 ± 0.58), favouring ProDisc-C (p<0.0001). Secondary surgical procedures significantly higher in ACDF group at 7 years (19/106 vs 7/103, p=0.0099).
Skeppholm et al 2015	Level 1	Randomized controlled multicenter superiority study comparing outcomes for ACDF (plate and autograft iliac crest bone) and Artificial Disc Replacement (Discover artificial disc).	Both groups significantly improved baseline NDI score at 2 year follow up (NDI mean baseline 63.1 compared to post-op 39.8, p<0.01), but no significant difference between

		153 patients recruited in Sweden from April 2007-May 2010 and randomized to ADR (n=83) or ACDF (n=70).<br><br>Baseline demographic data similar between groups: mean age (ADR 46.7±6.7, ACDF 47±6.9), male:female ratio (ADR 40:41, ACDF 33:37), Smoking Status (ADR 31%, ACDF 14%), BMI mean (ADR 26, ACDF 26), number of involved segments (ADR 58 single-level & 23 two-level, ACDF 50 single-level & 20 two-level).<br><br>Inclusion Criteria: age 25-60 years, refractory radiculopathy ≥3 months, one- or two-level cervical disease. Exclusion Criteria defined in study<br><br>Primary outcome: NDI. Secondary outcomes: VAS, HRQOL/EQ-5D, HAD, Dysphagia Short Questionnaire, sick leave, analgesic consumption, complication rates<br><br>Assessments preoperatively, then 4 weeks, 3 months, 1year and 2 years post-operatively. 2 year follow up: 137/153 (ADR=5, ACDF=9)	groups (NDI mean score ADR 39.1, vs ACDF 40.1)<br><br>Both groups improved from baseline EQ-5D and VAS scores at 2 years (p<0.01), but no difference between groups. No significant difference between groups in 2 year reoperation rates (ADR 9/83, ACDF 3/80).
Arnold et al 2016	Level 1	Prospective randomized controlled single-blinded non-inferiority multicenter FDA IDE trial comparing ACDF graft materials (autograft versus i-Factor bone graft). 319	Both groups had significant improvement from baseline NDI score (baseline i-Factor 50.6±13.2, vs autograft 52.7±14.4) at 12 months (i-Factor 28.75, autograft 27.40),

		patients recruited from June 2006-May 2013 across 22 cities (USA=19, Canada =3) and randomly assigned to autograft (n = 154) or i-Factor (n = 165). No significant difference between groups in baseline demographic data: age (i-Factor 47.7±9.8, Autograft 45.7±9.4), BMI (i-Factor 28.6±6.0, autograft 29.1±5.7), gender: (i-Factor 42.24% male, autograft 37.5% male), Smoking Status (i-Factor 20.5%, autograft 27.63%).<br><br>Inclusion Criteria: age 18-70 years, single-level radiographic DDD from C3-7, radiculopathy, VAS neck & arm scores >4, NDI >4, failure of 6 months non-operative treatment. Exclusion Criteria defined in study.<br><br>Primary outcomes: NDI, neurologic success endpoints, adverse events and radiographic fusion. Secondary endpoints: VAS neck and arm scores, SF-36v2, PCS, MCS, Odom scores.<br><br>Assessments pre-operatively and then postoperatively at 6 weeks, 3, 6, 9, 12, 18, and 24 months and annually thereafter. 24 month follow up rate: 87% (i-Factor 83.23%, autograft 91.45%).	*P*=0.0004, *P*<0.0001 respectively.<br><br>Both groups demonstrated significant improvement in neurological success rate at 12 months (i-Factor 93.71%, autograft 93.01%, *P*<0.0001), high fusion rate (i-Factor 88.97%, autograft 85.82%, *P*<0.0004). No significant difference in adverse events (i-Factor 83.64%, autograft 82.47%, *P*=0.8814). Overall success rate consisting of fusion, NDI, neurological success and safety success was higher in i-Factor group (i-Factor 68.75%, autograph 56.94%, respectively, *P*=0.0382).
Hisey et al 2016	Level 1	Prospective, randomized, controlled	No statistical significance between groups in

		multicenter non-inferiority design study comparing outcomes for Cervical Total Disc Replacement (TDR: Mobi-C) vs ACDF (allograft bone with plate). 245 patients recruited from 23 centers across USA into TDR (n=164) or ACDF (n=81).<br><br>Patient demographic data: age, BMI, male:female ratio, smoking status not reported.<br><br>Inclusion Criteria: symptomatic single-level DDD with radiculopathy or myeloradiculopathy from C3-7, disc height ≥3mm, ≥6 weeks failed conservative management. Exclusion Criteria defined in study.<br><br>Primary outcomes: NDI, VAS for neck & arm pain, SF-12, patient satisfaction, major complications, subsequent surgery, segmental ROM, ASD.<br><br>Assessments preoperatively and at 6 weeks, 3, 6, 12, 18, 24, 36, 48 and 60 months postoperatively. 5 year follow up rate: TDR 85.5% , ACDF 78.9%	NDI, VAS neck and arm pain, SF-12 scores, or major complication rates at 60 months<br><br>Subsequent surgery rates at 5 years (TDR: 3.0%, ACDF: 11.1%, $p<0.02$) and radiographic adjacent segment disease (TDR: 37.1%, ACDF: 54.7%, $p<0.03$) were significantly lower for TDR patients.
Lomeau et al 2016	Level 2	Prospective, randomized controlled non-inferiority design comparing outcomes for total disc arthroplasty (ProDisc-C) and ACDF (allograft bone & plating) with 84 month	VAS neck pain scores at baseline (ProDisc-C baseline 80.01 ± 10.8 vs ACDF baseline 73.86±16.6) compared to 7 years (ProDisc-C 11.67 ± 18.83 vs ACDF 27.98 ± 34.7),

		follow up. Single site results from a 13 center trial. 44 patients randomized to TDA (n= 22 + 19 via continued access) or ACDF (n= 22). Baseline patient demographics reported as similar, though mean age, BMI, male:female ratio not reported.<br><br>Inclusion Criteria: single level DDD between C3-7, radiculopathy, failure of at least 6 weeks non-operative management, NDI >15,<br><br>Exclusion Criteria defined in study.<br><br>Primary clinical outcomes: VAS for neck and arm pain, NDI, SF-36, MCS, PCS, adverse effects, and radiographic outcomes.<br><br>Assessments preoperatively and postoperatively at 6 weeks, 3, 6, 12, 18, 24, 36, 48, 60, 72, and 84 months. 84 month follow-up: 86% ACDF (19/22), 82% ProDisc-C (18/22), 34 % (7/19) of continued access ProDisc-C.	favouring ProDisc-C (*p*<0.007)<br><br>VAS arm pain scores (baseline ProDisc-C 74.6 ± 24.12 vs ACDF 71.7 ± 25.43) compared to 7 years (ProDisc-C 12.55 ± 23.36 vs ACDF 27.26 ± 35.71), favouring ProDisc-C (*p*=0.027)<br><br>NDI Scores at baseline (baseline ProDisc-C 53.95 ± 11.93 vs ACDF 53.64 ± 14.11) compared to 7 years (ProDisc-C 15.44 ± 19.59, ACDF 25.47 ± 23.96), favouring ProDisc-C (*p*=0.015)<br><br>Range of motion significantly reduced in ACDF 7 years (*p*<0.001).<br><br>No statistical significance between groups in SF-36. 7 year re-operation rate 27.3% (6/22) in ACDF vs 0% in ProDisc-C
Richter et al 2016	Level 3	Prospective comparative cohort study reporting results from Dynamic Cervical	Both groups showed significant improvement from their baseline scores at 12 months post-

		Implant (Paradigm Spine GmbH,) and ACDF (PEEK). 60 patients recruited between March 2009-September 2010 and randomized to ACDF (n=30) or DCI (n= 30). No differences in baseline patient demographics: age (DCI 44.1±8.8, SCDF 46±7.3), number of involved segments (DCI 73% one-level & 7% two-level, ACDF 70% one-level & 30% two-level), male:female ratio (DCI 16:10, ACDF 11:16), BMI not reported, Smoking Status not reported.<br><br>Inclusion Criteria: 1 or 2 level cervical DDD with radiculopathy. Exclusion Criteria defined in study<br><br>Primary outcomes: VAS-N,VAS-A, NPAD, EQ-5D scores, radiologic assessment (criteria defined in study)<br><br>Assessment 1 day pre-op and 3- & 12- months postoperatively. 12 month follow up: 88.3% (ACDF 26/30, DCI 27/30)	op:<br><br>VAS-N baseline (DCI 5.7±2.6 vs ACDF 6.9±2.4) compared to 12 month follow up (DCI 4.1±2.6, vs ACDF 3.3±2.6), though no significant difference between groups at 12 months (*p*=0.312)<br><br>VAS-A baseline (6.1± 2.4 vs ACDF 7.2±2.5) compared to 12 month follow up (DCI 2.5±2.5 vs ACDF 2.7±2.9), though no significant difference between groups at 12 months (*p*=0.826)<br><br>Also reported EQ-5D, NPAD, fusion rate, subsidence, implant dislocation occurrence.
Burkus et al 2017	Level 3	Prospectively administered and historically controlled study comparing rhBMP2 as osteoinductive protein for patients receiving ACDF vs TDR for symptomatic single-level cervical disease. N = 224 patients, investigational group with rhBMP2 compared	Subjective neck pain scores (preoperatively 15.8 vs 15.9 TDR group) vs 6 months (6.7 ACDF vs 5 TDR) vs 12 months (6.2 ACDF vs 5 TDR) vs 24 months (5.9 TDR vs 4.8 ACDF). Arm pain scores reported separately.<br><br>NDI for neck pain (53.5 ACDF vs 50.2 TDR

		with historical TDR groups (Data from PRESTIGE trial and BRYAN cervical TDR data). ACDF control patients received ATLANTIS cervical plate system (N = 486). Mean age 44.2 +/- 8.7, 251 females 235 males, 145 smokers, work status and litigation reported. Assessed at 6 12 and 24 months postoperatively. Operative time blood loss hospital stay times recorded.	baseline) vs 6 months (22.4 ACDF vs 16.9 TDR) vs 12 months (20.9 vs 16.7 TDR) vs 24 months (20.7 ACDF vs 15 TDR) with no standard deviations. Neurological success rates recorded at 6 12 and 24 months.
Pandey et al 2017	Level 3	Prospective single center study comparing ACDF (autologous iliac crest bone graft and anterior locking plate) and Cervical Total Disc Replacement (Prestige LP, Medtronic). 34 patients recruited between July 2012-April 2014 and randomized to ACDF (n=17) or CTDR (n=17). Baseline demographic data between groups: age (CTDR 29-57yrs, mean 39.7yrs, ACDF 31-55yrs, mean 39.7yrs), male:female ratio (CTDR 14:3, ACDF 13:4). BMI not mentioned. Mean operative time (116.4 minutes CTDR, 143 minutes ACDF). Inclusion Criteria: age 25-60 years, single level symptomatic disease between C3-7, failure ≥6 weeks of conservative management. Exclusion Criteria reported in study.<br><br>Primary outcome measures: NDI, VAS neck and arm pain, JOA scores, Nurick grade,	VAS mean score (baseline CTDR 7.52±0.30, ACDF 7.1±0.35) compared to 12 months (CTDR 1.47 ±0.3, ACDF 2.35 ±0.23). NDI score (baseline CTDR 58.52±4.74, ACDF 59.05±4.26) compared to 12 months (CTDR 13.64 ±1.78, ACDF 23.76± 2.36). Neck and Arm pain scores (baseline CTDR 48.94±1.76, ACDF 49.12±1.64) compared to 12 months (CTDR were 11.58±1.04, ACDF 17.64±1.42).<br><br>JOA, Nurick grade, and Odoms scoes reported. Significant difference between groups in range of motion favouring CTDR (*P* < 0.05). ACDF significantly higher adjacent segment disease (*p*<0.05)

		Odoms criteria, radiologic assessment<br><br>Assessments pre-operatively, 6 weeks, 3, 6, 12 months post op. Mean follow-up 18months.	
Razankovic et al 2017	Level 2	Randomized controlled trial comparing Discover Artificial Cervical Disc Replacement (n = 51) vs ACDF (n = 50) control. TDR 49% male vs ACDF 50% male, surgical levels are presented. Patients followed for 24 months. BMI and smoking status not reported.	VAS preoperatively (7.5 TDR and ACDF) vs 3 months (3.5ACDF vs 2.5 TDR) vs 6 months (3.5 ACDF vs 2.2 TDR) vs12 months (3.5 ACDF vs 2.2 TDR) vs 24 months 3.2 ACDF vs 2.2 TDR), standard deviations presented in table.<br>NDI (51 ACDF and TDR preoperatively) vs 3 months postoperative (20 ACDF vs 14 TDR) vs 6 months postoperative 19 ACDF vs 14 TDR) vs 12 months postoperative (19 ACDF vs 13 TDR) vs 24 months (20 ACDF vs 12 TDR), standard deviations reported in table. Complications reported separately.
Sasso et al 2017	Level 1	Prospective randomized single-center controlled FDA IDE trial comparing primary outcomes of NDI and VAS for neck & arm pain scores at 7- and 10- years post arthroplasty (Bryan Disc) vs. ACDF (allograft & single level plating, Medtronic).<br><br>47 patients enrolled and randomized to ACDF (n=25) vs Arthroplasty (n=22). Patient demographics reported to have no statistical significance between groups, though BMI,	Baseline NDI (Arthroplasty 50.45, vs ACDF 49.92) compared to 7 years (Arthroplasty 8.6 vs ACDF 21), and 10 years (Arthroplasty 8.05, ACDF 15.48) with significant between groups differences favouring Arthroplasty at 7 years (p=0.0138) and 10 years, p=0.0485).<br><br>Baseline VAS arm scores (Arthroplasty 7.77, vs ACDF 7.0) compared to 7 years (Arthroplasty 0.45, ACDF 1.88) and 10 years (Arthroplasty 0.84, vs ACDF 0.74), with

		average age, male:female ratio not mentioned.<br><br>Inclusion Criteria: single-level degenerative disc disease between C3-7 with 6 weeks of failed conservative management for either cervical radiculopathy or myelopathy, no prior cervical spine surgery, NDI>30, age ≥21 years. Exclusion Criteria reported in study.<br><br>Primary Outcomes: NDI & VAS.<br><br>Assessed pre-operatively, data from 7- and 10-years. 10 year follow up: 86.5% arthroplasty, 92% ACDF.	significant differences favouring arthroplasty at 7 years (*p*=0.0322),<br><br>Baseline VAS neck scores (Arthroplasty 7.18, vs ACDF 7.4) compared to 7 years (Arthroplasty 2.71 vs ACDF 0.9) and 10 years (Arthroplasty 1.26, vs ACDF 1.52) with significant difference favouring arthroplasty at 7 years (*p* = 0.0146), but not at 10 years (*p*=0.6958).
Arts et al 2017	Level 1	Single centre RCT, patients blinded for treatment. 2 year follow-up. Patients (N = 104 of 390 eligible enrolled) between 18 and 75 years of age with monoradiculopathy for a minimum of 8 weeks. Patients randomized to either Valeo C+CSC or PEEK cages for ACDF. All patients operated on by one of 2 authors of the study. Female 44% in silicon nitride group and 48% in PEEK. Mean age 53.3 in silicon nitride vs 49.4 in PEEK. 46% smokers in silicon group vs 40% in PEEK. Mean BMI 26.7 in silicon group vs 28.6 in PEEK.<br><br>Operative characteristics included operative time, blood loss, mean hospital stay, and	NDI (primary outcome measure) for neck and arm pain, silicon group NDI 42.6 +/- 17.1 baseline vs 42.8 +/- 14.9 in PEEK. At 12 months silicon group 24.4 +/- 20.6 vs PEEK 16.3 +/- 16.4.<br>SF-36 for physical function and bodily pain, VAS scale for neck and arm pain (VAS neck pain at 12 months 26.3 +/- 24.9 for silicon vs 22.5 +/- 26.8 for PEEK), Likert 7 point scale, radiological outcomes for subsidence (defined as > 3mm).

		complications (transient dysphagia 9 silicon vs 8 PEEK, cage subsidence 1 silicon 1 PEEK, infection with cage removal 1 silicon 0 PEEK, and adjacent level surgery 6 silicon vs 3 PEEK)	

Figure A1: VAS baseline scores compared to 6 week follow-up ($I^2 = 99.9\%$, $p < 0.001$)

Study name	Difference in means	Lower limit	Upper limit	p-Value
Porchet et al 2004	-1.500	-1.732	-1.268	0.000
Nabhan et al 2007	-4.600	-4.971	-4.229	0.000
Cosar et al 2008	-4.700	-5.007	-4.393	0.000
Nabhan et al 2009 Group 1	-0.370	-0.422	-0.318	0.000
Nabhan et al 2009 Group 2	-0.320	-0.368	-0.272	0.000
Murrey et al 2009	-3.800	-3.919	-3.681	0.000
Delamarter et al 2010	-3.600	-3.719	-3.481	0.000
Garrido et al 2010	-4.200	-4.441	-3.959	0.000
Ha et al 2013 Group 1	0.100	-0.457	0.657	0.725
Ha et al 2013 Group 2	0.600	-0.053	1.253	0.072
Kasliwal et al 2013 Group 1	-1.300	-1.671	-0.929	0.000
Kasliwal et al 2013 Group 2	-1.600	-1.941	-1.259	0.000
Kasliwal et al 2013 Group 3	-1.200	-1.518	-0.882	0.000
Coric et al 2013	-5.000	-5.214	-4.786	0.000
Phillips et al 2013	-4.200	-4.288	-4.112	0.000
Hisey et al 2016	-4.100	-4.237	-3.963	0.000
Pandey et al 2016	-3.000	-3.298	-2.702	0.000
	-2.524	-3.457	-1.591	0.000

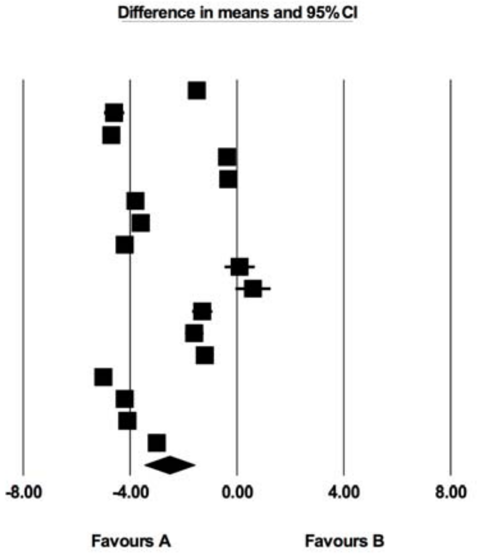

Meta Analysis

Figure A2: VAS baseline scores compared to follow-up beyond 48 months ($I^2 = 92.9\%$, $p<0.001$)

Study name	Difference in means	Lower limit	Upper limit	p-Value
Burkus et al 2010	-4.200	-4.713	-3.687	0.000
Garrido et al 2010	-4.400	-5.533	-3.267	0.000
Coric et al 2013	-7.000	-8.006	-5.994	0.000
Janssen et al 2015	-2.300	-2.866	-1.734	0.000
Hisey et al 2016	-4.800	-5.442	-4.158	0.000
Loumeau et al 2016	-4.600	-5.831	-3.369	0.000
Sasso et al 2017	-5.900	-7.056	-4.744	0.000
	-4.705	-5.829	-3.581	0.000

Statistics for each study

Difference in means and 95% CI

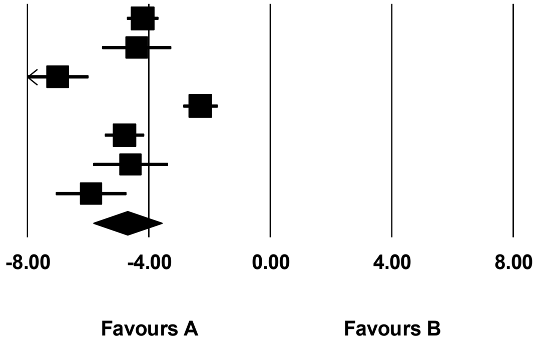

Meta Analysis

Figure A3: NDI scores at baseline compared to 6 week follow-up ($I^2 = 97\%$, $p < 0.001$)

Study name	Difference in means	Lower limit	Upper limit	p-Value
Porchet et al 2004	-35.000	-39.900	-30.100	0.000
Mummaneni et al 2007	-24.300	-25.991	-22.609	0.000
Heller et al 2009	-19.000	-20.851	-17.149	0.000
Murrey et al 2009	-26.000	-28.518	-23.482	0.000
Nabhan et al 2009 Group 1	-34.000	-43.165	-24.835	0.000
Nabhan et al 2009 Group 2	-32.000	-44.164	-19.836	0.000
Burkus et al 2010	-48.200	-50.501	-45.899	0.000
Garrido et al 2010	-21.000	-26.085	-15.915	0.000
Coric et al 2013	-25.600	-30.113	-21.087	0.000
Ha et al 2013 Group 1	-23.100	-25.207	-20.993	0.000
Ha et al 2013 Group 2	-31.400	-33.090	-29.710	0.000
Kasliwal et al 2013 Group 1	-18.000	-25.818	-10.182	0.000
Kasliwal et al 2013 Group 2	-22.000	-29.191	-14.809	0.000
Kasliwal et al 2013 Group 3	-18.000	-24.695	-11.305	0.000
Phillips et al 2013	-20.000	-21.857	-18.143	0.000
Hisey et al 2016	-28.000	-30.881	-25.119	0.000
Pandey et al 2016	-29.500	-35.788	-23.212	0.000
	-26.733	-30.881	-22.585	0.000

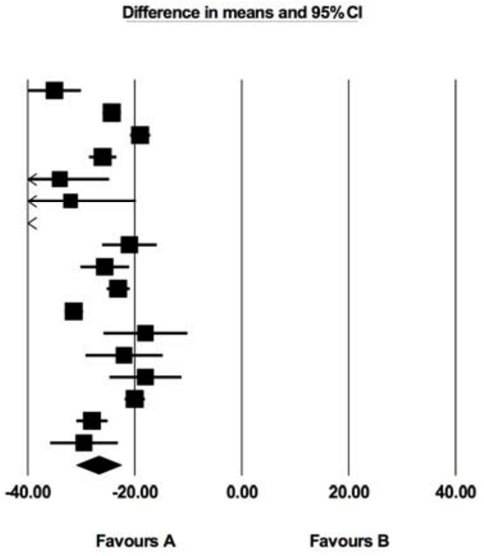

Meta Analysis

Figure A4: NDI baseline data compared to follow-up beyond 48 months ($I^2 = 91.0\%$, $p < 0.001$)

Study name	Statistics for each study			
	Difference in means	Lower limit	Upper limit	p-Value
Burkus et al 2010	-52.400	-56.019	-48.781	0.000
Garrido et al 2010	-32.000	-39.999	-24.001	0.000
Coric et al 2013	-39.300	-46.400	-32.200	0.000
Hisey et al 2016	-37.000	-41.532	-32.468	0.000
Loumeau et al 2016	-28.200	-36.896	-19.504	0.000
Sasso et al 2017	-34.500	-42.657	-26.343	0.000
	-37.609	-45.881	-29.337	0.000

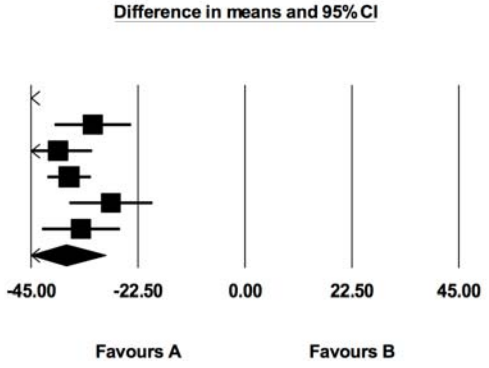

Meta Analysis
